# Loss of *Drosophila* Coq8 results in impaired survival, locomotor deficits and photoreceptor degeneration

**DOI:** 10.1186/s13041-022-00900-3

**Published:** 2022-02-09

**Authors:** Angelia J. Hura, Hannah R. Hawley, Wei Jun Tan, Rebecca J. Penny, Jessie C. Jacobsen, Helen L. Fitzsimons

**Affiliations:** 1 School of Natural Sciences, Massey University, Palmerston North, New Zealand; 2 Centre for Brain Research, School of Biological Sciences, The University of Auckland, Auckland, New Zealand

**Keywords:** coq8, COQ8A, Coenzyme Q10, Brain, Neuron, *Drosophila*, Neurodevelopment, Neurodegeneration, Photoreceptor, Mitochondria

## Abstract

*Coenzyme Q8A* encodes the homologue of yeast *coq8*, an ATPase that is required for the biosynthesis of Coenzyme Q10, an essential component of the electron transport chain. Mutations in *COQ8A* in humans result in CoQ10 deficiency, the clinical features of which include early-onset cerebellar ataxia, seizures and intellectual disability. The rapid advancement of massively parallel sequencing has resulted in the identification of more than 40 new mutations in *COQ8A* and functional studies are required to confirm causality and to further research into determining the specific mechanisms through which the mutations result in loss of function. To that end, a *Drosophila* model of Coq8 deficiency was developed and characterized to determine its appropriateness as a model system to further explore the role of Coq8 in the brain, and for functional characterisation of *Coq8* mutations. Pan-neuronal RNAi knockdown of *Coq8* was largely lethal, with female escapers displaying severe locomotor deficits. Knockdown of *Coq8* in the eye resulted in degeneration of photoreceptors, progressive necrosis and increased generation of reactive oxygen species. Reintroduction of wild-type *Coq8* restored normal function, however expression of human wild-type *COQ8A* exacerbated the eye phenotype, suggesting it was acting as a dominant-negative. This model is therefore informative for investigating the function of *Drosophila* Coq8, however human COQ8A mutations cannot be assessed as hCOQ8A does not rescue Coq8 deficiency.

**Supplementary Information:**

The online version contains supplementary material available at 10.1186/s13041-022-00900-3.

## Main text

Coenzyme Q10 (CoQ10, ubiquinone) is an essential component of the electron transport chain that shuffles electrons from complex I and complex II to complex III for mitochondrial respiration. Biosynthesis of CoQ10 in humans is orchestrated by a number of enzymes that are highly conserved across the animal kingdom [1]. One such enzyme is *Coenzyme Q8A* (*ADCK3*, *CABC1*), an atypical kinase of the UbiB protein kinase-like family, which localizes to the inner mitochondrial matrix [2] where it is activated by lipid precursors [3]. Its specific role in CoQ10 biosynthesis has not been fully elucidated, however it exhibits ATPase activity and is thought to couple hydrolysis of ATP to extraction of lipids into the aqueous matrix [3]. COQ8A also associates with other COQ biosynthetic proteins (COQ3-9), forming Complex Q [2, 4], and the presence of COQ8A is integral to its stability, as knockout of *COQ8A* in the mouse reduces the expression of all other COQ proteins in the complex in multiple tissues including the cerebellum and skeletal muscle [4].

Mutations in *COQ8A* in humans result in CoQ10 deficiency (OMIM: 612,016), the clinical features of which include early-onset cerebellar ataxia, seizures and intellectual disability [5–8]. The rapid advancement of massively parallel sequencing methodologies has resulted in the identification of more than 40 mutations in *COQ8A* [6–15] [16–22] and functional studies are required to confirm causality and to determine the specific mechanisms through which the mutations impact function. To that end, we developed and characterized a *Drosophila* model of Coq8 deficiency to further explore the role of Coq8 in the brain, and to assess the impact of loss of function *Coq8* mutations. *Drosophila Coq8* (*CG32649*) is orthologous to human COQ8A, sharing 53% identity/70% similarity in the C-terminal two-thirds of the protein (NCBI BLAST, accession NP_572836.1 vs NP_064632.2), which contains conserved sequence motifs that are central to the function of human COQ8A [3, 4] [23].

**Fig. 1:**
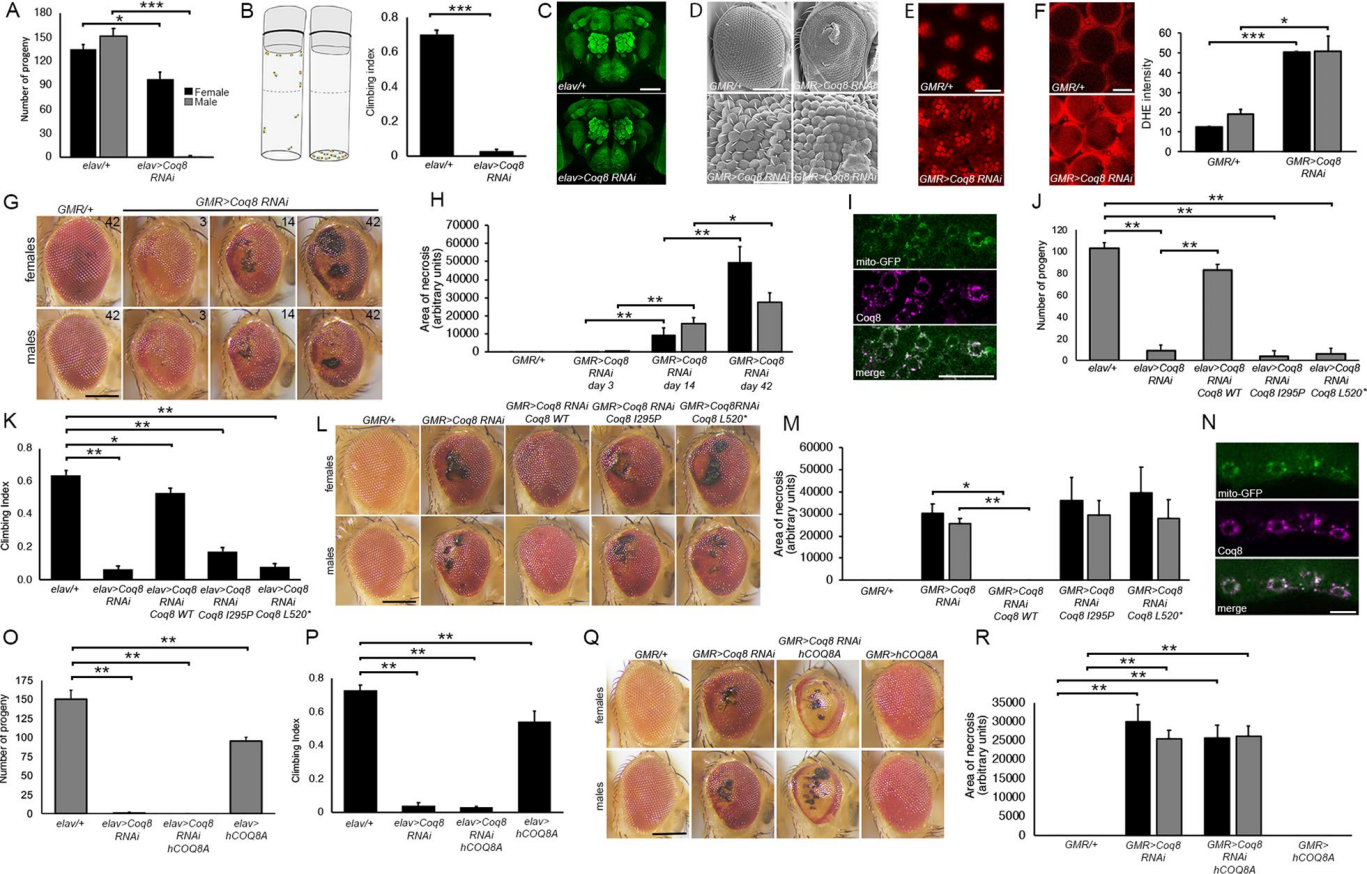
**A** Survival of male and female flies following knockdown of *Coq8* with *elav-GAL4*, which drives expression of *Coq8 RNAi* in all neurons. In all graphs, black bars indicate females and grey bars indicates males. *elav/* + indicates *elav-GAL4* crossed to the wild-type *w*^*1118*^ control. Flies were raised at 18 °C. The number of live male and female progeny were counted. Error bars indicate the standard error of three separate experiments. Female (*t*-test t_(4)_ = 3.585, *p = 0.0231); Male (*t*-test t_(4)_ = 16.2, ***p < 0.0001). **B** Flies were generated as described in **A**. The proportion of female flies (30/vial) to climb above 5 cm in 10 s was counted. Nine vials of flies were counted per genotype. (*t*-test t_(16)_ = 23.465, ***p < 0.0001). The schematic indicates the average climbing index of the *Coq8 RNAi* (CI = 0.03) and control (CI = 0.7) females. **C** Frontal confocal projections of whole brains processed for immunohistochemistry with anti-Bruchpilot (n = 6 brains/group). Scale bar = 100 μm. **D** *GMR-GAL4* males were crossed to *UAS-Coq8 RNAi* or *w*^*1118*^ females and the eyes of F1 progeny were analysed by light and scanning electron microscopy. Scale bar = 200 μm (top), 50 μm (bottom). **E** Nile red staining on dissected retinas. Scale bar 10 μm. **F** Dihydroethidium staining on dissected retinas. Scale bar = 10 μm. The fluorescence intensity was quantified (n = 3/group) Female (*t*-test t_(5)_ = 44.68, ***p < 0.0001); Male (*t*-test t_(5)_ = 3.3153, *p = 0.0211). **G** Flies were generated as described in **E**. Eyes were imaged by light microscopy at the indicated number of days post-eclosion. Scale bar = 200 μm. **H** The area of necrosis in each eye was quantified (n ≥ 8 eyes/group). (Female: ANOVA F(3,31) = 28.7 p < 0.0001; post-hoc Tukey’s test. Male: ANOVA F(3,32) = 27.12 p < 0.0001; post-hoc Tukey’s test, **p < 0.01, *p < 0.05). **I** Co-expression of mitoGFP and Myc-tagged *Drosophila* Coq8 with motor neuron driver *D42-GAL4*. Scale bar = 20 μm. J. *elav-GAL4* females were crossed to males of the indicated genotypes and the number of surviving adult male F1 progeny were counted (ANOVA F(4,10) = 51.95 p < 0.0001; post-hoc Tukey’s test, **p < 0.01). **K** The climbing ability of surviving females was assessed (ANOVA F(4,40) = 137.46 p < 0.0001; post-hoc Tukey’s test, **p < 0.01, *p < 0.05). **L** *GMR-GAL4* females were crossed to flies of the indicated genotypes and eyes were imaged by light microscopy 14 days post-eclosion. Co-expression of WT *Coq8* rescued the necrosis resulting from *Coq8* knockdown but *I295P* and L520* did not. **M** Quantification of necrosis in **L** (n ≥ 9 eyes/group) (Female: ANOVA F(4,43) = 7.73 p < 0.0001; post-hoc Tukey’s test. Male: ANOVA F(4,43) = 10.4 p < 0.0001; post-hoc Tukey’s test, **p < 0.01, *p < 0.05). **N** Co-expression of mitoGFP and human COQ8A with motor neuron driver *D42-GAL4* detected with anti-COQ8A. Scale bar = 12 μm. **O** *elav-GAL4* females were crossed to males of the indicated genotypes and the number of surviving adult male F1 progeny were counted (ANOVA F(3,8) = 133.22 p < 0.0001; post-hoc Tukey’s test, **p < 0.01). **P** The climbing ability of surviving females was assessed (ANOVA F(3,28) = 108.46 p < 0.0001; post-hoc Tukey’s test, **p < 0.01). **Q** Eyes of the indicated genotypes were imaged by light microscopy at 14 days post-eclosion. Scale bar = 200 μm. **R** Necrosis was quantified in the eyes of the indicated genotypes (n =  ≥ 9 eyes/group) (Female: ANOVA F(3,34) = 34.9 p < 0.0001; post-hoc Tukey’s test. Male: ANOVA F(3,34) = 77.4 p < 0.0001; post-hoc Tukey’s test, **p < 0.01)

We first investigated the importance of Coq8 to neuronal function in *Drosophila* via RNAi knockdown with the UAS/GAL4 system [24]. Pan-neuronal knockdown was lethal when flies were raised at 25 °C, however, when raised at 18 °C (at which temperature GAL4 is less active and thus there is reduced RNAi) some female flies survived (Fig. 1A), allowing characterization of the impact of Coq8 depletion on neuronal phenotypes. The female survivors displayed reduced movement with a tendency to accumulate at the bottom of the vial, therefore their locomotor function was assessed with the negative geotaxis assay [25]. Female flies displayed a profound climbing deficit, with only 3% climbing over 5 cm in comparison to 70% of controls which express GAL4 but do not carry the RNAi construct (Fig. 1B).

We next sought to examine whether the behavioral changes were associated with changes in brain morphology and function. The gross architecture of the brain was assessed via immunohistochemistry with anti-Bruchpilot, which labels the synaptic neuropil and allows visualization of the ultrastructure of the brain [26]. No gross morphological deficits were observed (Fig. 1C) and examination of individual sections did not indicate increased vacuolation.

We next examined the impact of *Coq8* depletion on photoreceptor development and integrity and observed a profound impairment in ommatidial patterning, with detachment of ommatidia and misaligned bristles (Fig. 1D). Each ommatidium develops with a characteristic asymmetric trapezoid arrangement of photoreceptors as visualized by Nile Red, which stains lipids in the rhabdomeres [27] (Fig. 1E top). When Coq8 was reduced, this regular arrangement was disrupted (Fig. 1E bottom). As *COQ8A* mutations have been associated with oxidative stress [2], dihydroethidium staining [28] was performed on retina, which showed an increase in reactive oxygen species (Fig. 1F). Necrotic patches were also evident on the surface of the eye (Fig. 1G) and a progressive increase in necrosis was observed, with area of the necrotic tissue increasing in size 111-fold in males and 490-fold in females from day 3 to 42 (Fig. 1H,  p < 0.0001).

Expression of Myc-tagged wild-type Coq8 in neurons resulted in robust expression in the adult brain and appropriate localization to mitochondria as indicated by colocalization with mito-GFP [29] (Fig. 1I). Co-expression of *Coq8* rescued the male lethality resulting from *Coq8* knockdown (Fig. 1J), as well as the climbing deficits (Fig. 1K) and necrosis (Fig. 1L, M), confirming the specificity of the RNAi. Two mutants of *Coq8* (I295P and L520*) were generated that were based on corresponding human mutations (p.Leu277Pro and c.1506 + 1G > A) that we previously identified in a sibling pair [7]. Unlike the complementation observed on reintroduction of WT *Coq8*, neither mutant rescued the lethality (Fig. 1J), the climbing deficits (Fig. 1K) or impaired eye development (Fig. 1M). Immunohistochemical analyses revealed that expression of I295P was reduced and L520* expression was not detected (Additional file 3). We next examined whether human wild-type *COQ8A* could rescue *Coq8* knockdown. The *hCOQ8A* cDNA was expressed and localized appropriately to mitochondria (Fig. 1N). However, it was unable to rescue male lethality (Fig. 1O), climbing deficits (Fig. 1P) or necrosis (Fig. 1Q, R), in fact expression of hCOQ8A in a wild-type background impaired survival and climbing. Moreover, hCOQ8A also appeared to act as a dominant-negative in the eye, resulting in a more severe phenotype than *Coq8* knockdown alone, with smaller eyes displaying a complete loss of ommatidial integrity and pigmentation, resulting in a glossy phenotype (Fig. 1Q), which has been previously observed in mutants for mitochondrial function [30]. Coq8 functions as a dimer, thus it is possible that hCOQ8A interferes with Coq8 function, the impact of which is exacerbated when Coq8 is already depleted.

Taken together, these data show that Coq8 is essential for survival in *Drosophila*, and is required for normal locomotor function and survival of photoreceptors. This model of Coq8 deficiency can be used for investigating the function of *Drosophila* Coq8. The lack of rescue by hCOQ8A suggests that *Drosophila* Coq8 has additional functions to hCOQ8A and warrants further investigation.

## Supplementary Information

**Additional file 1.** Materials and Methods.

**Additional file 2.** References 16 to 30.

**Additional file 3: Figure S1.** Expression of Myc-tagged WT *Coq8*, *Coq8* I295P and *Coq8* L520* in the *Drosophila* brain. Anti-Myc (green) detects the Myc-tagged proteins, and anti-Bruchpilot detects the neuropil marker nc82 (magenta). All genotypes were generated by crossing *elav-GAL4* females to males carrying each indicated *UAS-Coq8* transgene and to the *w*^*1118*^ control (*elav-GAL4/* +*).* Representative images of frontal confocal projections of whole brains (n = 6/group) are shown. Robust expression of WT Coq8 was observed across the brain, with localization to the cytoplasm of neurons as seen in the magnified image in the inset. Expression of I295P was much reduced, and L520* was not detected, with staining at a similar level to the control. Scale bar = 100 μm.

## Data Availability

The datasets supporting the conclusions of this article are included within the article.
