# Supplementary material for: Loss of Drosophila Coq8 results in impaired survival, locomotor deficits and photoreceptor degeneration
Source: Mol Brain. 2022 Feb 9;15:15. doi: 10.1186/s13041-022-00900-3 (PMC8827264; doi:10.1186/s13041-022-00900-3)
Supplement: Supplementary file 1 — Additional file 1. Materials and Methods. [file 13041_2022_900_MOESM1_ESM.docx]

**Materials and Methods**

Fly stocks and crosses

All flies were raised on standard medium on a 12-hour light/dark cycle and maintained at a temperature of 25°C unless otherwise indicated. *P{w[+mC]=GAL4-ninaE.GMR}12* (*GMR-GAL4*), *P{w[+mW.hs]=GawB}elav[c155]* (*elav-GAL4*) and *w[1118]; P{w[+mW.hs]=GawB}D42, P{w[+mC]=UAS-mito-HA-GFP.AP}3 e[1]/TM6B, Tb[1] (D42, mito-GFP)* were obtained from the Bloomington *Drosophila* Stock Center. *w^1118^; P{KK107966}VIE-260B* (*UAS-Coq8* RNAi, VDRC ID 110801) was obtained from the Vienna *Drosophila* Resource Center. *Drosophila Coq8* constructs were synthesised from nucleotides 160-2145 of *Coq8*, NCBI reference NM_132608, with a Myc tag and codon optimized for *Drosophila.* *Coq8-Myc* was cloned into *Not*I and *Xba*I of pUASTattB by Genscript. The I295P and L520* mutants were generated by Genscript via site directed mutagenesis. They were based on corresponding human mutations (p.Leu277Pro and c.1506+1G>A) that we previously identified in a sibling pair (7). The c.1506+1G>A mutation destroys a splice donor site, resulting in truncation of the protein. Leu277Pro is a missense mutation within a conserved KxGQ motif (KLGQ to KPGQ) found in the substrate-binding pocket of the protein (23). The human COQ8A construct was synthesized from nucleotides 116-2059 of *hCOQ8A*, NCBI reference NM_020247.5, codon optimized for *Drosophila* and cloned into *Not*I and *Xba*I of pUASTattB by Genscript. Transgenic flies were generated by Genetivision using the VK37 docking site at (2L) 22A3. Female flies carrying the appropriate GAL4 driver were crossed to males flies harbouring UAS-transgene or UAS-RNAi construct. In the F1 progeny, expression of GAL4 in the target tissue resulted in activation of transgene expression on binding of GAL4 to the UAS.

Negative Geotaxis Assay

The Negative Geotaxis assay was used to assess locomotor function. Thirty female flies from each genotype were anesthetised and placed in flat bottom vials. The flies were gently banged to the bottom of the vial, and then the number of flies that climbed above 5 cm in 10 seconds were counted. This number was divided by the total number of flies to generate a Climbing Index.

Immunohistochemistry

Adult flies were prefixed in PFAT-DMSO (4% paraformaldehyde, 1X PBS, 0.1% Triton-X and 5% DMSO) for 2 hours at room temperature before being washed in PBST (1X PBS, 0.5% Triton-X). Brains were then dissected in PBST and post-fixed for 20 mins. Following 4 x 5 min washes in PBST, brains were blocked for 2 hours with immunobuffer (5% Normal Goat Serum, 1X PBST) then incubated overnight at room temperature in primary antibody in immunobuffer, washed 3x in PBST, followed by incubation in secondary antibody overnight at 4 ̊C. Brains were washed in PBST before being mounted in Antifade (0.02% N-propyl gallate, 90% glycerol in 1XPBS). Confocal microscopy was carried out with a Leica SP5 DM6000B Scanning Confocal Microscope and images processed with Adobe Photoshop and ImageJ. The following antibodies were used: anti-Bruchpilot nc82 (1:50, Developmental Studies Hybridoma Bank), anti-Myc 9E10 (1:50, Developmental Studies Hybridoma Bank) for detection of *Drosophila* Coq8, anti-COQ8A ab221193 (1:500, Abcam) for detection of human COQ8A and anti-GFP ab290 (1:5,000, Abcam) for detection of mito-GFP.

Scanning Electron Microscopy

Flies were placed in Karnovsky fixative (3% glutaraldehyde, 2% formaldehyde in 0.1 M phosphate buffer, pH 7.2, plus Triton X-100) for eight hours at room temperature before being washed with phosphate butter (0.1 M, pH 7.2) and dehydrated using a graded ethanol series. The Polaron E3000 series II critical point drying apparatus was used to dry the samples using liquid CO_2_. Heads were mounted onto aluminium stubs and coated with gold (Bal-Tec SCD 050 Sputter Coater) then viewed and imaged using an FEI Quanta 200 Environmental Scanning Electron Microscope at an accelerating voltage of 20 kV.

Light Microscopy

Eyes were imaged on an Olympus SZX16 Microscope using CellSens Dimension (Olympus) Software. Images were collected at 110x magnification every 10 μm through the eye and optical sections were stacked with the Auto-Blend layers function of Adobe Photoshop. Necrosis was quantified by outlining the area of necrosis and determining the number of pixels with the histogram tool.

Nile red staining

Adult flies were prefixed in PFA-DMSO (4% paraformaldehyde, 1X PBS and 5% DMSO) for one hour at room temperature before being washed in 1X PBS. Retinas were then dissected in PBS and post-fixed for 30 mins in 4% PFA in 1X PBS. Following 3 x 5 min washes in PBS, retinas were incubated overnight in 0.002 mg mL^-1^ Nile red (Invitrogen) diluted in PBS, then washed 3 x 5 min in PBS. Retinas were mounted in 70% glycerol and imaged on a Leica SP5 DM6000B Scanning Confocal Microscope. Images were processed with Adobe Photoshop.

Dihydroethidium staining

Flies were briefly anaesthetized with CO_2_ before dissecting for retinas in cold Schneider media. Retinas were incubated for 15 min in 20 μM dihydroethidium (Invitrogen) at room temperature wrapped in foil. Following 3 x 5 min washes with Schneider media, retinas of all genotypes were all mounted in 1X PBS under the same coverslip to minimise variation in signal intensity. Retinas were immediately imaged on a Leica SP5 DM6000B Scanning Confocal Microscope using the same laser power and gain setting. Mean fluorescent intensity of each sample was measured using ImageJ.
