# Supplementary material for: Loss of Drosophila Coq8 results in impaired survival, locomotor deficits and photoreceptor degeneration
Source: Mol Brain. 2022 Feb 9;15:15. doi: 10.1186/s13041-022-00900-3 (PMC8827264; doi:10.1186/s13041-022-00900-3)
Supplement: Supplementary file 2 — Additional file 2. References 16 to 30. [file 13041_2022_900_MOESM2_ESM.docx]

References 16 to 30

16. Liu G, Ma D, Li J, Luo C, Sun Y, Zhang J, et al. A novel COQ8A missense variant associated with a mild form of primary coenzyme Q10 deficiency type 4. Clin Biochem. 2020;84:93-8.

17. Liu YT, Hersheson J, Plagnol V, Fawcett K, Duberley KE, Preza E, et al. Autosomal-recessive cerebellar ataxia caused by a novel ADCK3 mutation that elongates the protein: clinical, genetic and biochemical characterisation. J Neurol Neurosurg Psychiatry. 2014;85(5):493-8.

18. Malgireddy K, Thompson R, Torres-Russotto D. A novel CABC1/ADCK3 mutation in adult-onset cerebellar ataxia. Parkinsonism Relat Disord. 2016;33:151-2.

19. Mutlu-Albayrak H, Kirat E, Gurbuz G. Childhood-onset autosomal recessive ataxias: a cross-sectional study from Turkey. Neurogenetics. 2020;21(1):59-66.

20. Shalata A, Edery M, Habib C, Genizi J, Mahroum M, Khalaily L, et al. Primary Coenzyme Q deficiency Due to Novel ADCK3 Variants, Studies in Fibroblasts and Review of Literature. Neurochem Res. 2019;44(10):2372-84.

21. Sun M, Johnson AK, Nelakuditi V, Guidugli L, Fischer D, Arndt K, et al. Targeted exome analysis identifies the genetic basis of disease in over 50% of patients with a wide range of ataxia-related phenotypes. Genet Med. 2019;21(1):195-206.

22. Wirth T, Tranchant C, Drouot N, Keren B, Mignot C, Cif L, et al. Increased diagnostic yield in complex dystonia through exome sequencing. Parkinsonism Relat Disord. 2020;74:50-6.

23. Stefely JA, Reidenbach AG, Ulbrich A, Oruganty K, Floyd BJ, Jochem A, et al. Mitochondrial ADCK3 employs an atypical protein kinase-like fold to enable coenzyme Q biosynthesis. Mol Cell. 2015;57(1):83-94.

24. Brand AH, Perrimon N. Targeted gene expression as a means of altering cell fates and generating dominant phenotypes. Development. 1993;118(2):401-15.

25. Barone MC, Bohmann D. Assessing neurodegenerative phenotypes in Drosophila dopaminergic neurons by climbing assays and whole brain immunostaining. J Vis Exp. 2013(74):e50339.

26. Wagh DA, Rasse TM, Asan E, Hofbauer A, Schwenkert I, Durrbeck H, et al. Bruchpilot, a protein with homology to ELKS/CAST, is required for structural integrity and function of synaptic active zones in Drosophila. Neuron. 2006;49(6):833-44.

27. Liu L, Zhang K, Sandoval H, Yamamoto S, Jaiswal M, Sanz E, et al. Glial lipid droplets and ROS induced by mitochondrial defects promote neurodegeneration. Cell. 2015;160(1-2):177-90.

28. Owusu-Ansah E, Banerjee U. Reactive oxygen species prime Drosophila haematopoietic progenitors for differentiation. Nature. 2009;461(7263):537-41.

29. Rizzuto R, Brini M, Pizzo P, Murgia M, Pozzan T. Chimeric green fluorescent protein as a tool for visualizing subcellular organelles in living cells. Curr Biol. 1995;5(6):635-42.

30. Liao TS, Call GB, Guptan P, Cespedes A, Marshall J, Yackle K, et al. An efficient genetic screen in Drosophila to identify nuclear-encoded genes with mitochondrial function. Genetics. 2006;174(1):525-33.
