# Supplementary figures and images for: Loss of Drosophila Coq8 results in impaired survival, locomotor deficits and photoreceptor degeneration
Source: Mol Brain. 2022 Feb 9;15:15. doi: 10.1186/s13041-022-00900-3 (PMC8827264; doi:10.1186/s13041-022-00900-3)

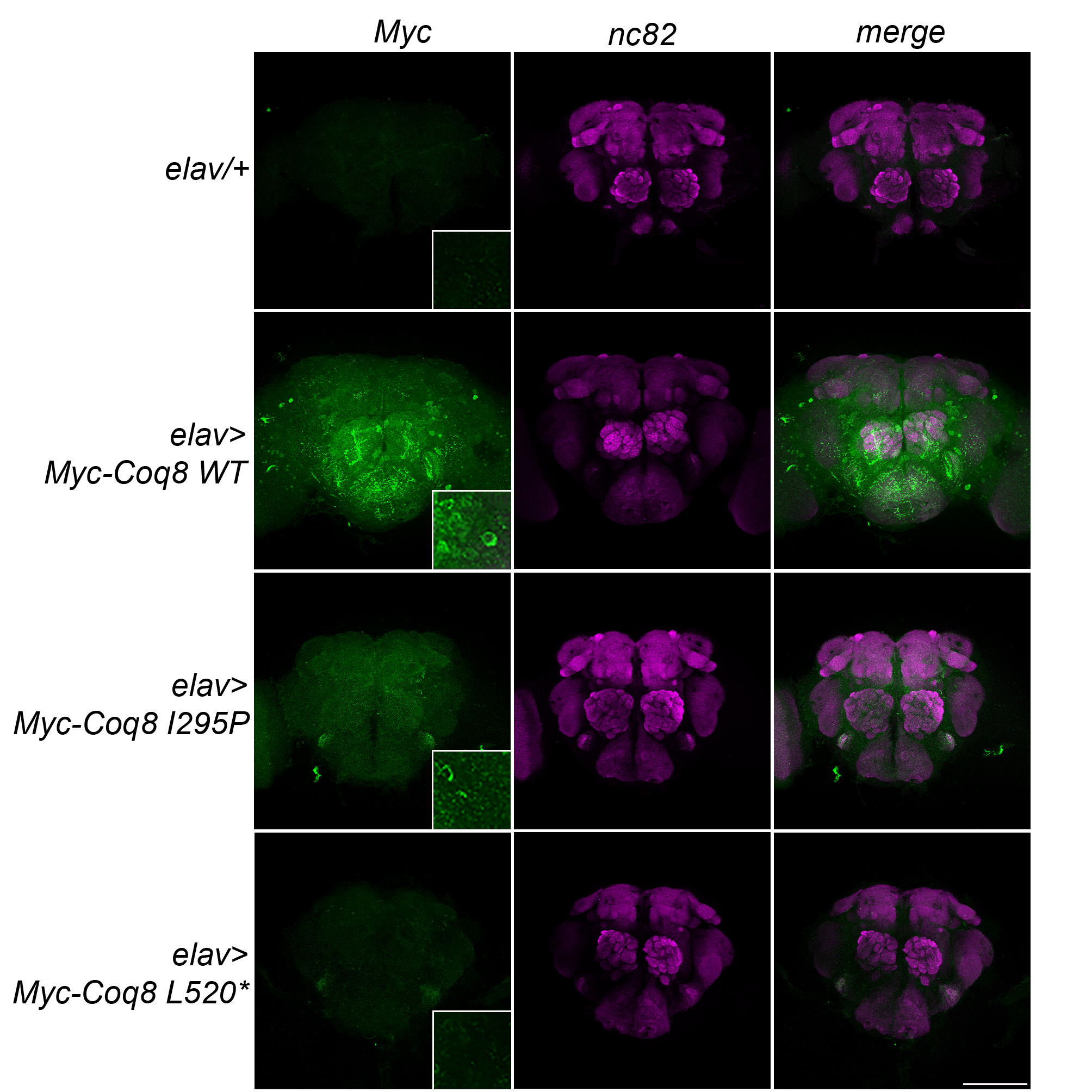

Supplement: Supplementary file 3 — Additional file 3: Figure S1. Expression of Myc-tagged WT Coq8, Coq8 I295P and Coq8 L520* in the Drosophila brain. Anti-Myc (green) detects the Myc-tagged proteins, and anti-Bruchpilot detects the neuropil marker nc82 (magenta). All genotypes were generated by crossing elav-GAL4 females to males carrying each indicated UAS-Coq8 transgene and to the w1118 control (elav-GAL4/ +). Representative images of frontal confocal projections of whole brains (n = 6/group) are shown. Robust expression of WT Coq8 was observed across the brain, with localization to the cytoplasm of neurons as seen in the magnified image in the inset. Expression of I295P was much reduced, and L520* was not detected, with staining at a similar level to the control. Scale bar = 100 μm. [file 13041_2022_900_MOESM3_ESM.tif]
